# Enhancement of Pig Embryonic Implants in Factor VIII KO Mice: A Novel Role for the Coagulation Cascade in Organ Size Control

**DOI:** 10.1371/journal.pone.0008362

**Published:** 2009-12-21

**Authors:** Anna Aronovich, Dalit Tchorsh, Elias Shezen, Chava Rosen, Yael Klionsky, Sivan Cohen, Orna Tal, Uri Martinowitz, Helena Katchman, Smadar Eventov-Friedman, Ninette Amariglio, Jasmine Jacob-Hirsch, Gideon Rechavi, Yair Reisner

**Affiliations:** 1 Department of Immunology, Weizmann Institute of Science, Rehovot, Israel; 2 The Israel National Hemophilia Center, Sheba Medical Center, Tel Hashomer, Israel; 3 Cancer Research Center, Sheba Medical Center, Tel Hashomer, Israel; University of Southern California, United States of America

## Abstract

Very little is known about the mechanisms that contribute to organ size differences between species. In the present study, we used a mouse model of embryonic pig tissue implantation to define the role of host Factor VIII in controlling the final size attained by the implant. We show here that pig embryonic spleen, pancreas, and liver all grow to an increased size in mice that are deficient in the Factor VIII clotting cascade. Similar results were obtained using the transplantation model after treatment with the low molecular weight heparin derivative Clexane which markedly enhanced transplant size. Likewise, enhanced size was found upon treatment with the direct thrombin inhibitor Dabigatran, suggesting that organ size regulation might be mediated by thrombin, downstream of Factor VIII. Considering that thrombin was shown to mediate various functions unrelated to blood clotting, either directly by cleavage of protease-activated receptors (PARs) or indirectly by cleaving osteopontin (OPN) on stroma cells, the role of PAR1 and PAR4 antagonists as well as treatment with cleaved form of OPN (tcOPN) were tested. While the former was not found to have an impact on overgrowth of embryonic pig spleen implants, marked reduction of size was noted upon treatment with the (tcOPN). Collectively, our surprising set of observations suggests that factors of the coagulation cascade have a novel role in organ size control.

## Introduction

Despite great progress in the molecular dissection of embryogenesis, the mechanisms controlling the size of tissues and organs remain a mystery. In general, it is thought that extrinsic control mechanisms are associated with nutrition or systemic growth signaling factors, such as those operating through the insulin/PI3K or the TOR pathways, while intrinsic mechanisms are likely linked to patterning morphogens and apoptosis-signaling complexes, as well as to stem cell numbers[1]–[4]. Some of the early insights in this area of investigation came from transplantation experiments in which infant rat hearts or kidneys transplanted into adult rats grew and attained the size of an adult organ[5]. Similar results were also found by Metcalf et al. in early transplantation experiments with fetal mouse organs. For example, when multiple fetal mouse thymus glands were transplanted into an adult mouse, each was found to grow to its normal adult size[6]. However, when the same experiment was performed using fetal spleens, the total mass of the transplanted spleen tissue attained the mass of only a single normal adult spleen, suggesting that the growth of these fetal tissues is limited by extrinsic factors, outside the spleen[7].

Very recently, we defined optimal ‘windows’ of gestation of human and pig embryonic precursor tissues that afford optimal growth and development of liver, pancreas and spleen upon implantation into NOD-SCID mice[8]. Our studies enabled not only a rational selection of fetal tissue for transplantation but, among many parameters, also provide a system in which to manipulate and study organ size control. Using this system, we have now found, surprisingly, that a difference in the functionality of one gene in the recipients, namely coagulation Factor VIII, has a major impact on the final size attained by different embryonic pig tissues upon transplantation into mice. Interrogation of other factors along the coagulation cascade that are triggered by Factor VIII, such as Factor Xa and thrombin, suggests that thrombin, which is the terminal enzyme of the hemostatic system, is likely to be the actual mediator of this novel checkpoint of organ size control. Furthermore, our ability to reverse the enhancement of implant size by treatment with the cleaved form of OPN that is released upon treatment with thrombin, strongly indicates that thrombin likely exerts its regulatory activity through interaction with OPN on stroma cells, as previously shown for maintenance of the blood tissue size under normal steady state hoemostasis[9], [10].

## Results

### The Role of Factor VIII in Defining Organ Size, Following Implantation of Pig Embryonic Tissues

**Figure 1:**
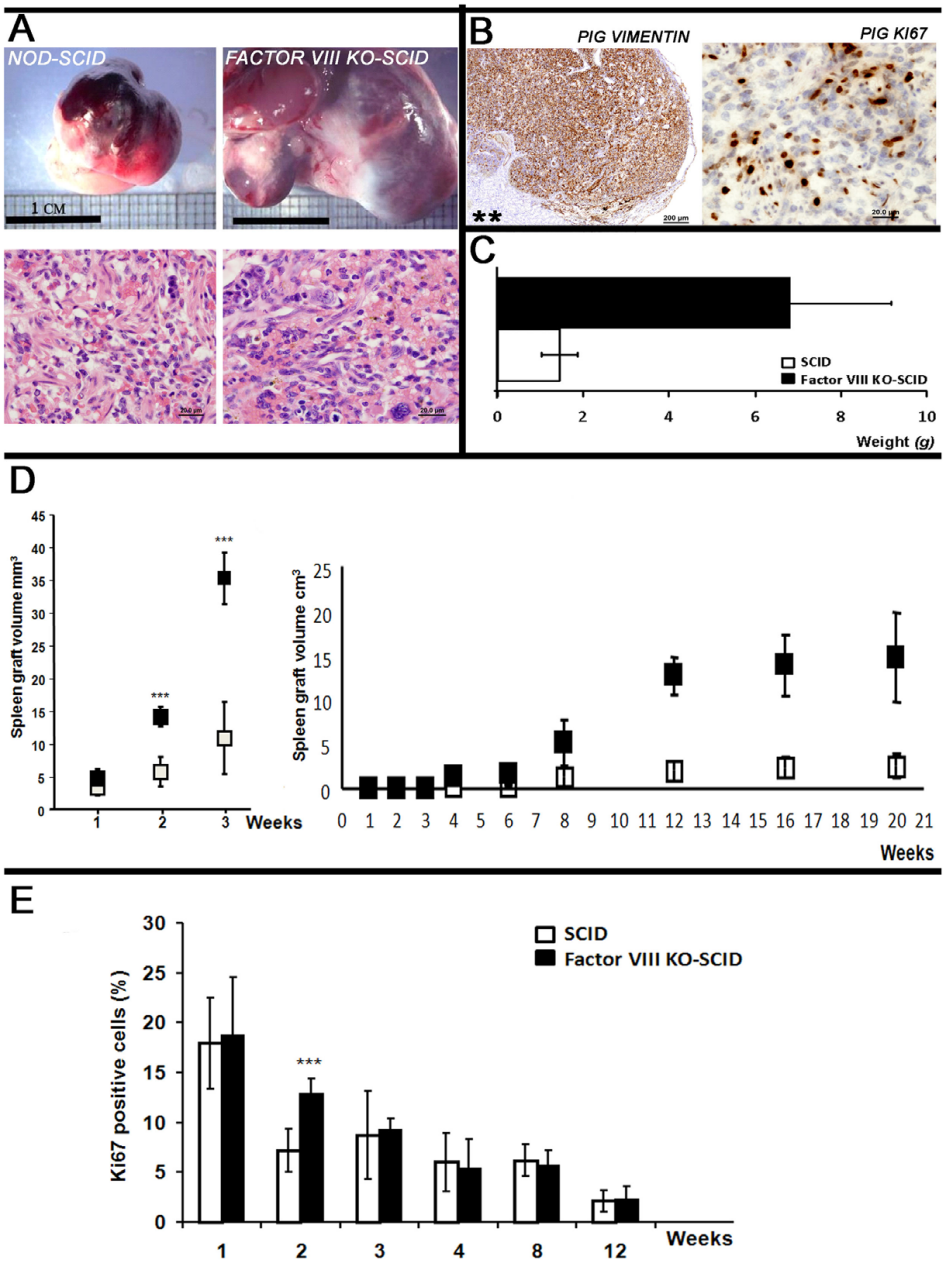
Growth enhancemnt of E42 embryonic spleen implants in Factor VIII KO-SCID mice. (A) Upper panel: Macroscopic view at 3 months post transplant of E-42 spleen grafts implanted into NOD-SCID or Factor VIII KO SCID recipients. Lower panel: Representative H&E staining revealing similar morphological patterns in NOD-SCID (left) and in Factor VIII KO SCID (right) mice. (B) Pig mesenchymal components stained with anti-vimentin (V9) and proliferating pig cells stained with anti-ki67 within the implant growing in Factor VIII KO recipients are shown in brown. Asterisk points to mouse kidney not stained by these pig specific antibodies. (C) Average weight of E42 spleen grafts at 3 months post transplant in the two types of recipients, (n = 20). (D) Graft volume of the implants in the two recipient groups during the first 3 weeks post transplant and over a 5 months follow-up period. (E) Percentage of dividing ki67 positive cells per square mm in the growing implants.

We have previously shown that implantation of pig embryonic spleen precursor tissue can correct hemophilia in factor VIII KO NOD-SCID mice by providing the necessary Factor VIII[11]. While performing this study, we observed that the size of the implanted spleen of these hemophilic mice was significantly larger compared to that growing in non-hemophilic control mice. Fig. 1A shows a typical oversized pig E42 spleen implant grown for 12 weeks in Factor VIII KO SCID recipient, versus the size of a similar implant grown in a Factor VIII expressing SCID recipient. The total average weight of a series of implants in Factor VIII KO SCID mice was 6.78±2.16gr versus 1.46±0.82gr in the normal SCID (Fig. 1C,), suggesting that the absence of Factor VIII enhanced spleen weight by a factor of 4 (p<0.05). Histological examination of the growing spleen implants (Fig. 1A, lower panel) revealed normal growth, development and vascularization patterns, comparable to those found in the corresponding Factor VIII wild type mice, ruling out potential induction of a malignant process. Furthermore, as shown by immuno-staining with pig specific antibodies for vimentin and ki67 (Fig. 1B), the bulk of the growing implant in factor VIII KO recipients was of pig origin.

Weekly follow up of E42 spleen transplants revealed that disparity in transplant size between NOD-SCID and Factor VIII KO-SCID mice occurs as early as 2 weeks post transplantation (Fig. 1D, left). Further tracking of E42 spleen transplants showed exponential growth until 12 weeks post transplantation, whereby the final implant size has been reached without further growth during an additional 2 months follow-up period (Fig. 1D, right). This conclusion was also supported by morphmetric quantitative examination of pig dividing cells, stained by pig specific anti-ki67 antibody. As can be seen in Fig. 1E, 2 weeks post transplantation a significant increase in the frequency of dividing cells was found in Factor VIII KO-SCID compared to NOD-SCID mice. However, beginning on the 3rd week a gradual decrease in dividing cells is observed, reaching a low level at 12 weeks post transplantation in Factor VIII KO recipients (2.7±1.3) similarly to that found in the non-hemophilic recipients (2.1±1.2). Thus, early post transplant events during the initial two weeks are critically affected by the lack of factor VIII in the recipient mice.

**Figure 2:**
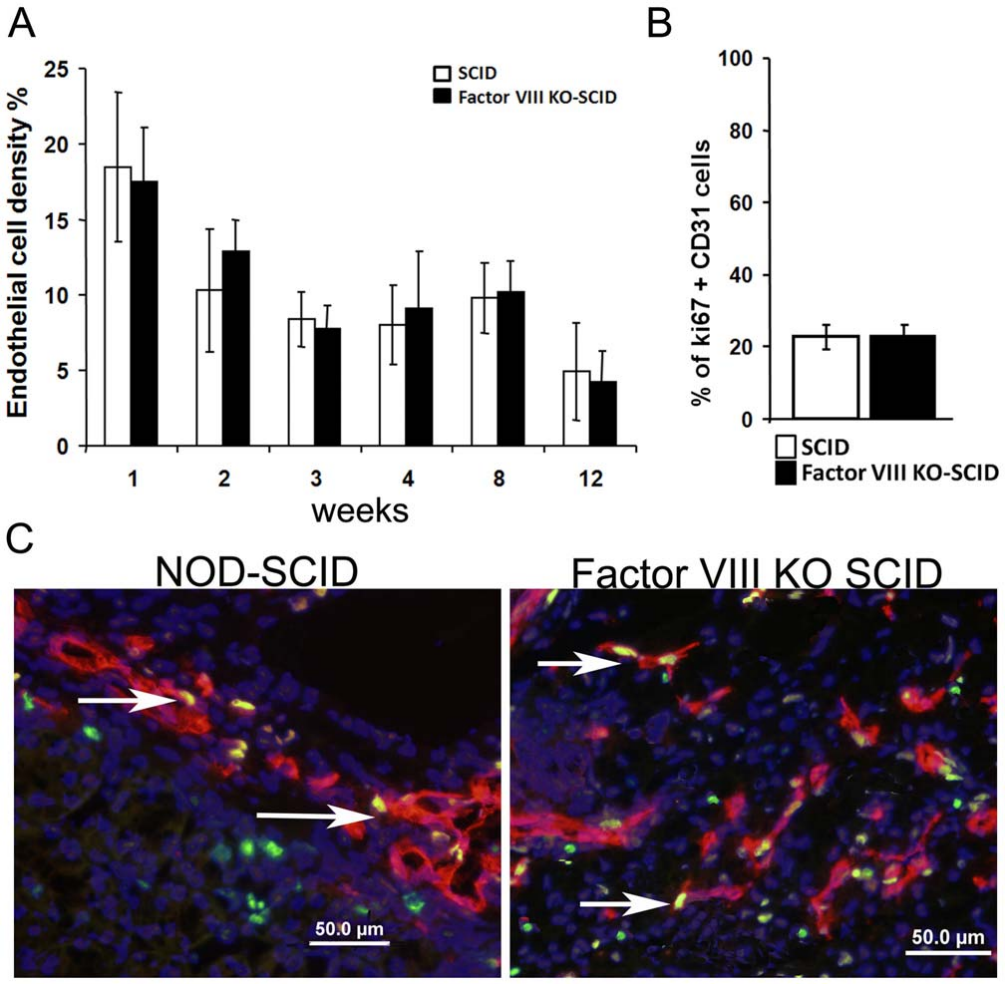
Angiogenesis pattern for the E42 spleen implants growth. (A). Morphometric analysis of endothelial density based on CD31 positive cells following implantation of E-42 spleen grafts into NOD-SCID or Factor VIII KO SCID recipients. (B) Quantitative evaluation of the numbers of proliferating endothelial cells per total number of nucleated proliferating cells in a square mm of the growing tissues. Analysis was performed at 2 weeks post transplantation. (C) Representative double staining of CD31+ endothelial cells (red) and Ki67+ proliferating cells (green). Nuclei are in stained in blue. Arrows outline proliferating endothelial cells (yellow) in the grafts.

Considering that angiogenesis is an absolute requirement for the implant growth we also performed morphometric analysis of CD31 positive cells in the implant. As can be seen in Fig. 2A, similar density of endothelial cells was found in both types of recipients, with no significant enhancement in the hemophilic recipients at any time point post transplantation. This observation implies that the enhanced growth of transplants in Factor VIII KO-SCID mice is not due to angiogenesis advantage. Furthermore, quantitative evaluation of the numbers of proliferating endothelial cells per total number of nucleated proliferating cells did not reveal a significant difference between hemophilic 21.9±1.9% and non-hemophilic 22.4±2.3% recipients (Fig. 2B).

**Figure 3:**
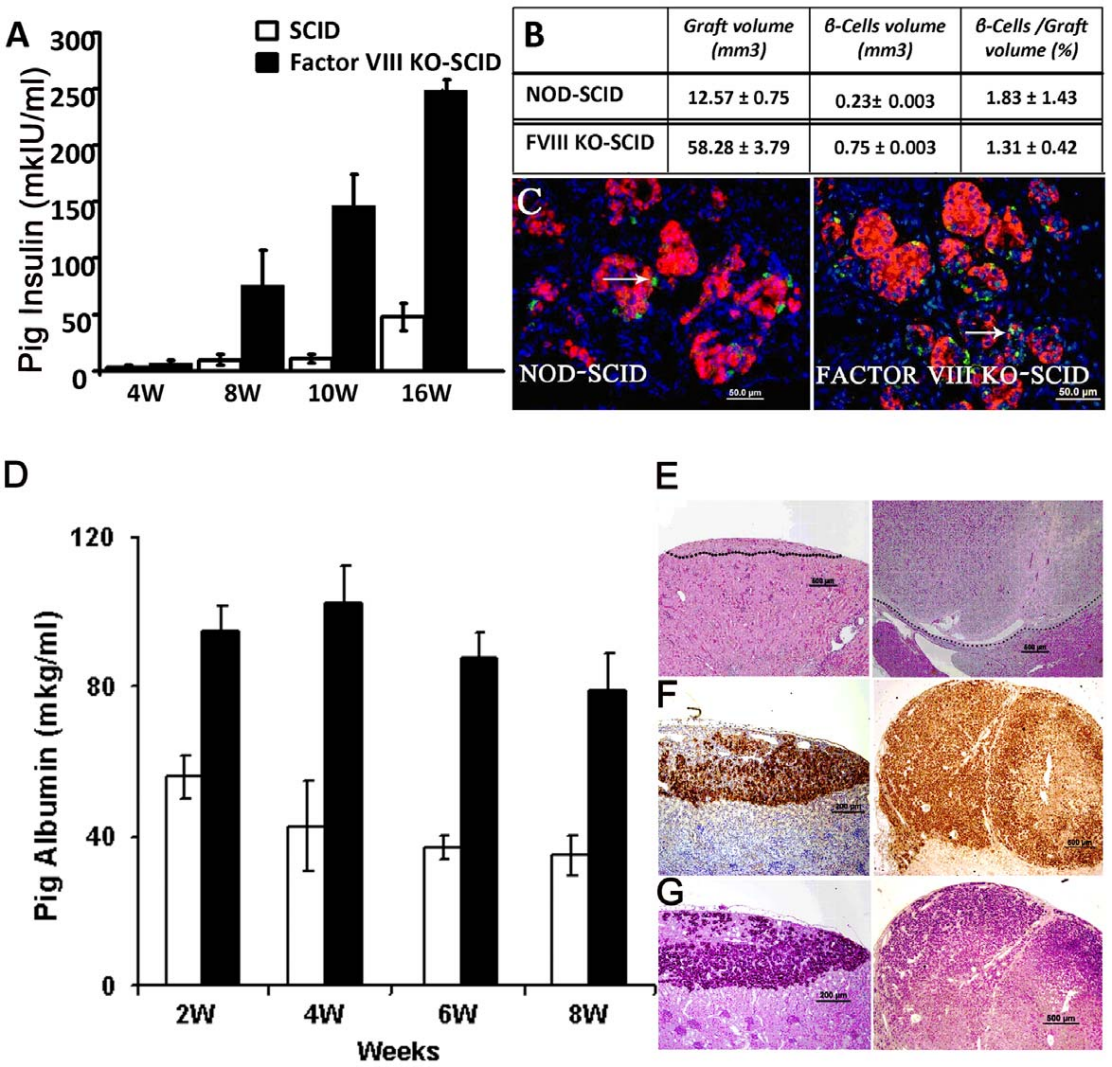
Enhancement of pancreas and liver size and function in Factor VIII KO- SCID mice. (A) Pig insulin serum levels following implantation of E42 pig pancreas into Factor VIII KO SCID and NOD-SCID mice. Data represent averages of four independent experiments (P<0.005). (B) Morphometric analysis of E42 pancreas implants growth. Both recipients were evaluated for graft volume (left column) and beta cells volume (middle column) for pig insulin positive beta cell volume (right column), 3 months after transplantation (n = 5). The specific difference in volume was not associated with a significant increase in β cell number. (C) Normal histological findings following E42 pig pancreas transplantation under the kidney capsule of NOD-SCID and Factor VIII KO-SCID mice. Pig insulin appears red, while glucagon is stained green. (D) Pig albumin serum levels following implantation of E42 pig liver precursor tissue into Factor VIII KO SCID and NOD-SCID mice. Data represent three independent experiments (P<0.05). Illustration of increased growth and retained functionality of the liver grafts in Factor FIII KO-SCID recipient, demonstrated by H&E staining (E), and immunohistological staining of pig albumin (F), and PAS (G).

To define whether our observation on the growth of embryonic spleen is also applicable to other tissues, we used the same experimental system and evaluated growth of pig embryonic liver and pancreas precursor tissue harvested at previously defined optimal ‘windows’, namely E28 and E42, respectively[8]. The growth of pancreatic tissues was determined in the SCID recipient mice by monitoring blood levels of pig insulin using a specific ELISA, previously shown to correlate with implant size[8]. As can be seen in Fig. 3A, pig insulin blood levels were markedly enhanced in Factor VIII KO SCID mice compared to Factor VIII positive SCID mice, reflecting a growth advantage in the Factor VIII KO recipients. Since these differences in insulin levels might be attributed to differences in functionality of β-cells rather than to the total β -cell number, we determined the total volume of the implants as well as the fraction of β-insulin positive cells out of the total volume (Fig. 3B), using morphometric analysis. Enhancement of pig pancreas size measured by this parameter was found to closely parallel the difference in insulin blood levels. Thus, the total volume of insulin positive cells was 0.75±0.003 mm^3^ in Factor VIII SCID, versus 0.23±0.003 mm^3^ in non-hemophilic SCID recipients, respectively, suggesting an overall enhancement of implant size by at least a factor of three (p<0.05).

As previously reported, E42 pancreatic implants into NOD-SCID mice are predominantly composed of endocrine tissue with minimal exocrine activity[11]. Only a minimal number of exocrine cells were detected in the E42 graft 3 months after transplantation, while most of the cells were of the endocrine lineage. Similar architecture of the endocrine compartment of the growing pig pancreas was noted in Factor VIII KO NOD-SCID recipients (Fig. 3C). Thus, the significant size difference was not associated with a difference in the architecture of the growing implant.

A significant enhancement, by at least a factor of two, of implant size was also found upon implantation of embryonic pig liver in Factor VIII KO SCID mice, as evaluated by ELISA for pig blood albumin levels (Fig. 3D). As in the case of spleen and pancreas, despite its enhanced growth in Factor VIII KO hosts, the growing pig liver exhibited similar architecture in both types of recipients (Fig. 3E–G).

### RAG−/−Factor VIII KO Mice Also Exhibit Enhanced Implant Size Following Transplantation of Pig Embryonic Tissues

It is possible that the observed enhancement of growth in Factor VIII KO NOD- SCID mice is due to a potential genetic abnormality affecting some other aspect of their metabolism. Prkdc^scid^ (commonly referred to as SCID) mice carry a spontaneous mutation in chromosome 16. In NOD-SCID mice, this mutation was backcrossed onto the NOD/ShiLt background. Hemophilic (Factor VIII KO) mice are homozygous for a targeted, X chromosome-linked mutant allele, inserted using a neo cassette, which was used to disrupt exon 16 of the Factor VIII gene. The Factor VIII KO mice on a NOD-SCID background that we used were derived by crossing these two strains[11]. However, in order to rule out any potential artifacts specific to this strain combination, we introduced the Factor VIII KO mutation into a different SCID mouse, namely, SCID induced by knockout of the RAG gene. RAG^−/−^ mice were produced by inserting a targeted mutation on chromosome 2, resulting in a “non-leaky” severe combined immune deficiency[12]. A new RAG−/− FVIII KO colony was therefore established to confirm our findings on the effect of FVIII KO on organ size.

**Figure 4:**
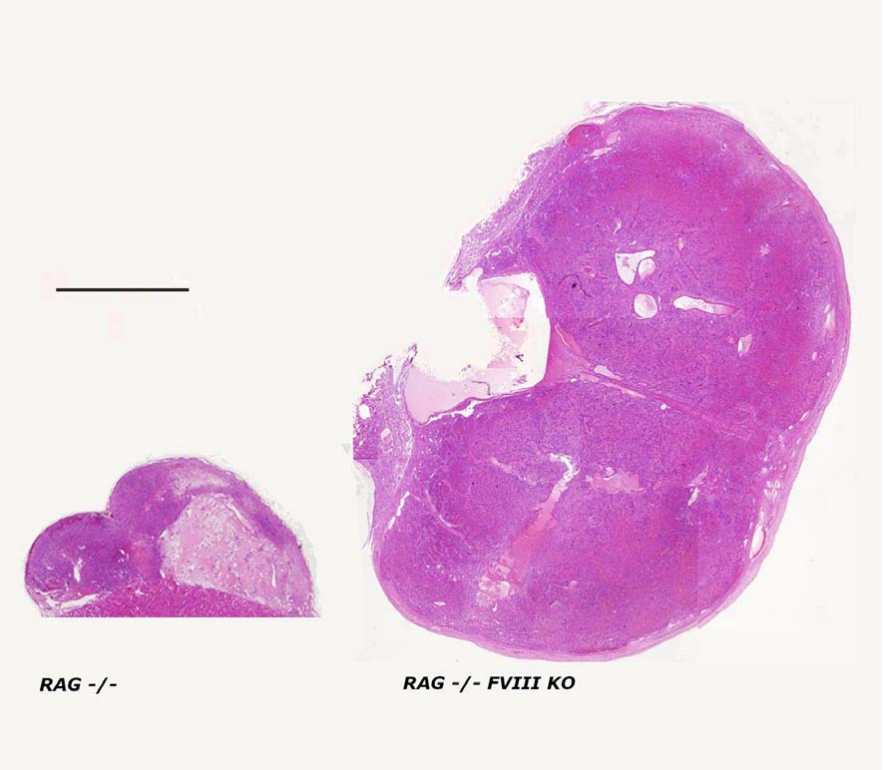
H&E staining of an E42 pig spleen implant 12 weeks following transplantation. Oversized spleen implant is shown in RAG−/− FVIII KO compared to its non-hemophilic RAG−/− counterpart. Bar indicates 2 mm.

As can be seen in Fig. 4, RAG−/− FVIII KO recipients of an E42 pig spleen implant develop an oversized spleen implant, compared to their non-hemophilic RAG−/− counterparts.

Thus, the Factor VIII KO mutation was associated with enhanced growth in this strain combination, as well, ruling out potential artifacts due to confounding factors other than Factor VIII deficiency.

### Embryonic Mouse Spleen, Pancreas or Liver Implanted in Factor VIII KO SCID Mice Exhibit No Size Enhancement

The enhancement of organ size following implantation of pig embryonic spleen, pancreas and liver could be related to early events associated with graft accommodation and formation of vasculature, which may potentially differ in Factor VIII KO versus non-hemophilic SCID recipients, in a manner that is independent of the origin of the donor tissue. To address this possibility, similar implantation experiments were repeated using embryonic tissues of mouse origin. In contrast to the pig implants, no differences in organ size were found between SCID Factor VIII KO and non-hemophilic SCID recipients following implantation of mouse E15 gestational age tissues. Spleen grafts of mouse origin exhibited an similar size, 3 months post transplantation of 8.98±2.55 in SCID and 9.12±1.14 SCID Factor VIII KO recipients. Similar results were obtained for mouse E16 pancreas and liver transplants (data not shown). Furthermore, no differences in size were found following transplantation of embryonic mouse tissues obtained from hemophilic donors into Factor VIII KO-SCID versus non-hemophilic SCID mice. Thus, our results suggest that while the final size of heterologous embryonic pig implants is affected by the presence or absence of mouse Factor VIII, embryonic mouse implants attain their final size regardless of the host environment.

The lack of any effect on mouse embryonic transplants, regardless of the expression of Factor VIII by the host or the donor, suggests that the role of Factor VIII is likely limited to a checkpoint of excessive growth, which operates upon implantation of tissue from a larger animal, such as the pig. More specifically, it is possible that Factor VIII or one of its derivatives might interfere with the activity of a putative survival factor, and thereby may have an important role in defining the maximum tolerable tissue mass. Thus, if we assume that mouse and pig embryonic implants are endowed with stem cell pools of different sizes prior to transplantation, they are likely to attain different organ size upon completion of growth and differentiation in the mouse recipients. Consequently, in hemophilic mice lacking the putative inhibitory activity (i.e. overgrowth checkpoint) mediated by Factor VIII, the size of the pig implants is likely to be larger, while mouse implants growing to their expected [mouse] size will not be subject to Factor VIII control.

### The Role of Downstream Members of the Coagulation Pathway in Organ Size Control

**Figure 5:**
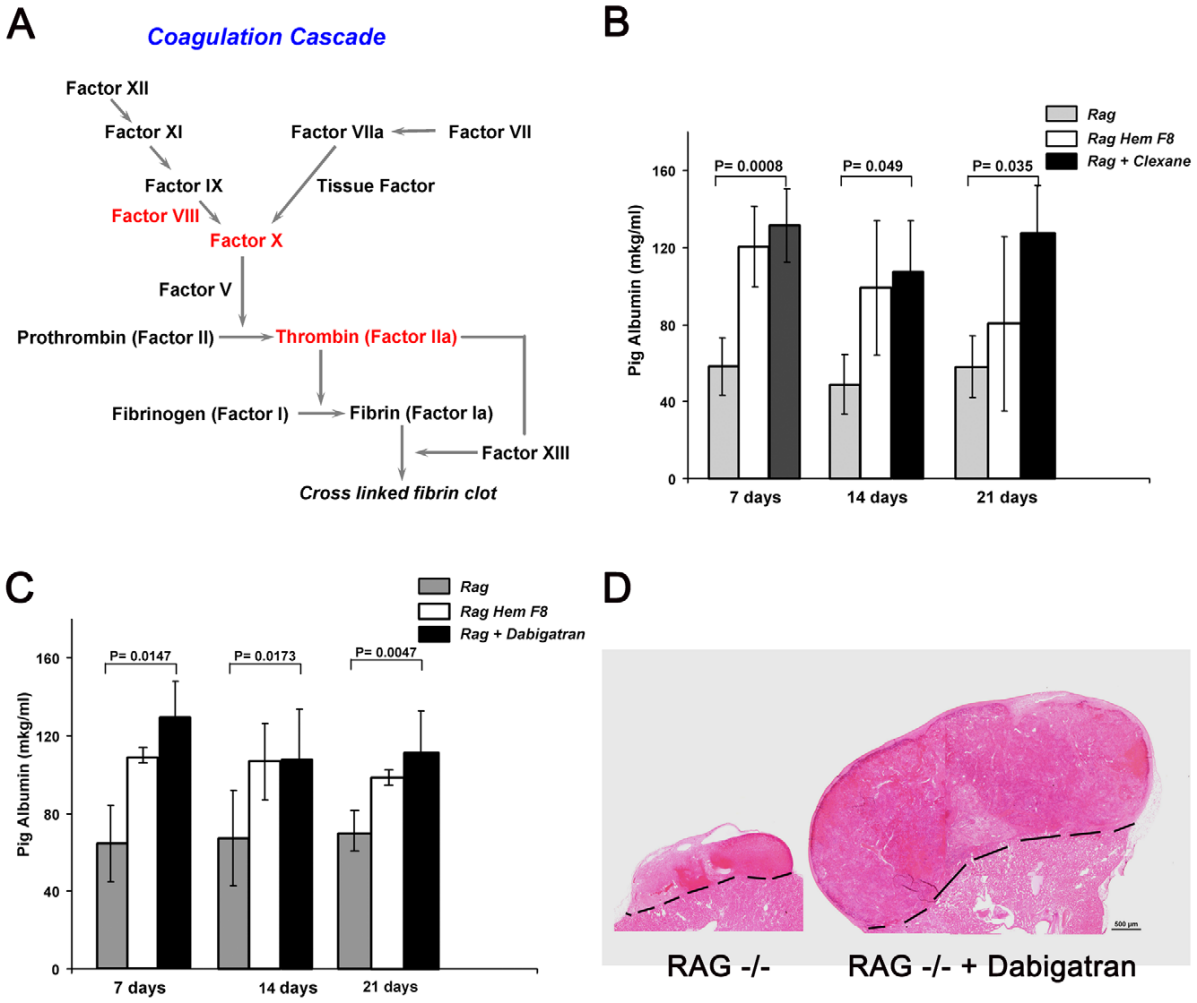
Direct involvement of coagulation cascade factors in enhancement of growth of pig embryonic transplants. A. Coagulation cascade scheme. B. Enhancement of embryonic pig liver growth by Clexane administration in Rag−/− mice C. Enhancement of embryonic pig liver growth by Dabigatran administration in Rag−/− mice. Pig albumin serum levels, detected by specific ELISA, are shown at 7, 14 and 21 days after implantation of E42 pig liver precursor tissue in the presence or absence of Dabigatran administration. The results are compared to those obtained in hemophilic Rag−/− factor VIII KO mice. D. E42 pig spleen transplant with and without Dabigatran administration in hemophilic Rag−/− factor VIII KO mice.

Our present findings indicate a role for Factor VIII in organ size control, through an unknown mechanism. This intriguing role could be mediated directly by this molecule or through one of the other coagulation factors activated along the cascade triggered by Factor VIII. Factor Xa is activated by Factor VIII and, in turn, thrombin is activated by Factor Xa (Fig. 5A). Thus, either of these downstream factors could potentially mediate the observed size enhancement in our assays.

One approach to dissect the role of these factors was to use the anti-coagulant, Clexane (a low molecular weight heparin derivative) which blocks Factor Xa and, to a lesser degree, thrombin. We evaluated organ size in the pig liver transplantation model in the presence or absence of Clexane administration. Implantation of pig embryonic liver provides a rapid assay, as growth can be monitored by the appearance of pig albumin (detectable by specific ELISA) in the mouse serum as early as 7 days post transplant. As can be seen in Fig. 5B, Clexane administration in non-hemophilic recipients induced marked enhancements of pig albumin blood levels on days 7, 14 and 21 post transplant compared to control recipients, not receiving Clexane.

Since Clexane could potentially block not only Factor Xa, but also inhibits thrombin to some extent, further analysis was performed using a more specific inhibitor, namely, Dabigatran, which blocks only thrombin. As can be seen in Fig. 5C, Dabigatran administration led to marked enhancement of pig embryonic liver implants, similar to that exhibited by Clexane. Representative E42 spleen transplant with and without Dabigatran administration is shown in Fig. 5D. Those results identify thrombin as a potential direct player in the enhancement of pig transplants growth.

### The Role of PAR's and OPN Cleavage by Thrombin

**Figure 6:**
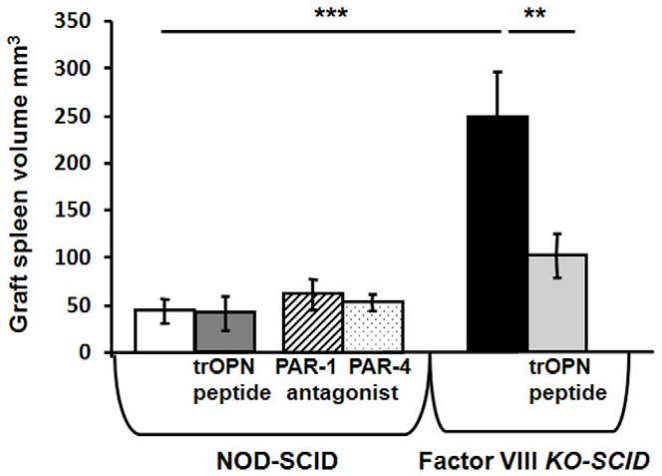
The role og PARS and OPN cleavage by thrombin in defining size of E42 pig embryonic spleen implants. E42 pig spleen tissue was implanted into NOD-SCID and FactoorVIII KO NOD -SCID mice and final size of the implant was defined 4 weeks after transplantation. To define the effect of PARs cleavage by thrombin, PAR-1 and PAR-4 antagonists were administered to NOD-SCID recipients as described in Methods. To evaluate the role of OPN cleavage, trOPN was administered to Factor VIII NOD-SCID recipients as described in Methods. Each group comprised 7 mice. Enhancement of growth of E42 spleen tissue in Factor VIII NOD-SCID mice compared to NOD-SCID mice was statistically significant (t test, p<0.005)). This enhancement was significantly inhibited upon treatment with trOPN (p<0.05).

Thrombin is a serine protease that transforms fibrinogen into fibrin and activates blood platelets. However in addition to its role in coagulation, multiple effects on a variety of cell types including endothelial cells, vascular smooth muscle cells (VSMC), monocytes, T lymphocytes and fibroblasts have been suggested[13]–[15]. Cellular effects of thrombin are mediated by PARs, members of the G protein-coupled receptors that carry their own ligand which remains cryptic until unmasked by proteolytic cleavage by thrombin[15], [16]. Thrombin can also affect cells indirectly by cleaving OPN which is a secreted, multifunctional glycoprotein that exists either as a full-length molecule or as proteolytically cleaved fragments. A unique N-terminal fragment of OPN (trOPN), produced by thrombin cleavage has previously been shown to be present in plasma and milk and may have physiologically distinct roles[17]-[19]. In particular, It has been previously demonstrated by Nilsson et al. [20] that trOPN acts as a negative regulator of both human and mouse hemopoietic stem and progenitor cell (HSC/HPC) proliferation and differentiation in vitro. Recently this group had expanded their study and has shown that in the bone marrow OPN exists predominantly in its thrombin-cleaved forms. Notably, the N-terminal cleavage fragment binds to both alpha9beta1 and alpha4beta1 integrins, which are expressed on HSC/HPC and by that plays a key role in the attraction, retention, regulation, and release of hematopoietic stem and progenitor cells to, in, and from their BM niche [21]. Thus, it was of interest to define whether thrombin enhances implant size through PAR signaling or via its effect on OPN. In order to investigate thrombin's receptors PAR signaling we have used specific PAR-1 and PAR-4 antagonists which were previously described in the literature as potent agents in blocking thrombin activation[22]–[25]. As can be seen in Fig. 6, treatment of non-hemophilic recipients with PAR1 or PAR4 antagonists did not reveal any enhancement of pig spleen implants. In contrast, treatment of Factor VIII KO recipients of pig spleen implants with trOPN exhibited a significant inhibition of implant size comparable to that found in the non-hemophlic recipients, suggesting that the regulatory role of thrombin is mediated through trOPN, cleaved by thrombin from OPN.

## Discussion

Organ size control during embryonic development or in tissue regeneration, involves a fine balance between cell growth, proliferation and death, maintained by extrinsic and intrinsic factors[1]–[4].

Our assay using heterologous embryonic transplantation provides an additional tool in the study of growth control, in that it enables investigation of the cross-talk between extrinsic host factors and intrinsic mechanisms relevant to size control in the implant. In particular, this assay enables pinpointing the role of different extrinsic genes by using mutated or KO host mice. Thus, we demonstrate, for the first time, that host Factor VIII plays a critical role as an extrinsic factor in controlling the final size of pig-derived organs.

The lack of any effect on mouse embryonic transplants, regardless of whether the host or the donor is derived from a hemophilic or non-hemophilic mouse, suggests that the role of Factor VIII is likely limited to oversize growth checkpoint that operates upon implantation of tissue from a larger animal, such as the pig. Thus, if we assume that mouse and pig embryonic implants are endowed with stem cell pools of different size prior to transplantation, they are likely to attain different organ sizes upon completion of growth and differentiation in the mouse recipients. Consequently, in hemophilic mice lacking the potential inhibitory activity (i.e. overgrowth checkpoint) mediated by Factor VIII, the size of the pig implants is likely to be larger, while mouse implants growing to their normal expected size will not exhibit excessive growth, and therefore will not be subject to Factor VIII control.

Further interrogation of other factors downstream of Factor VIII in the coagulation cascade revealed that thrombin is probably the Factor VIII downstream factor that actually mediates regulation of oversize control in our models.

**Figure 7:**
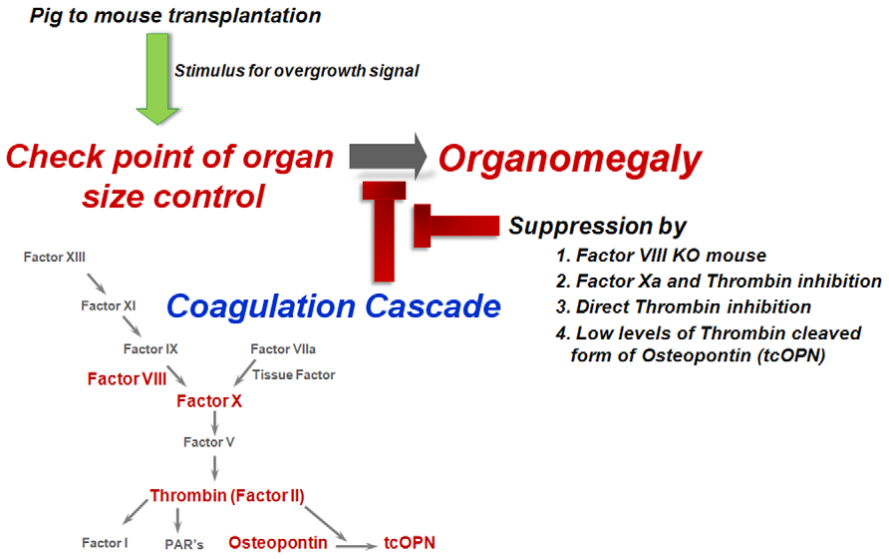
Overgrowth stimulus regulation by factors in the coagulation cascade.

Thus, as outlined schematically in Fig. 7, interference with steady state levels of factors of the coagulation cascade, such as occurs in Factor VIII KO mice or upon blockade of Factor Xa or thrombin, also overrules this checkpoint, thereby leading to oversized organs.

The suggestion that thrombin is a likely candidate for the actual inhibitory activity leading to oversize control, is supported by its terminal position in the hemostatic system, acting downstream of Factor VIII and Factor Xa.

The possibility that thrombin is indeed the bridge between the coagulation system and the checkpoint of organomegaly, is in line with a substantial body of literature demonstrating involvement of thrombin in various functions unrelated to blood clotting. Thus, thrombin is capable of signaling through PARs[26], which are expressed throughout the body and known to be involved in vascular responses[27], embryonic development[28], and malignancies[29]. Interestingly, several of these activities, including platelet activation and stimulation of tumor cell proliferation[30], [31], are associated with promotion of growth, which is in opposition to our suggestion that inhibition of thrombin leads to massive overgrowth. Indeed, we found that treatment with PAR1 and PAR4 antagonists did not affect implants size in our assay.

While PAR signaling pathways might control stem/progenitor cells directly, another major pathway could potentially be mediated non-autonomously by stromal cells in the stem cell niche, through the cleavage of osteopontin (Spp1, OPN) by thrombin. Osteopontin (Spp1, OPN) is a highly acidic phosphoprotein with pleiotropic effects, including regulation of inflammation, cell adhesion and angiogenesis. OPN has recently been demonstrated to be a pivotal molecule in limiting the size of the HSC pool[32]. Interestingly, it has been shown that the thrombin-cleaved form of OPN promotes the quiescence of HSCs by exerting a profound suppression of proliferation of HSCs, without inducing apoptosis[9]. OPN is a negative regulatory element of the stem cell niche that limits the size of the stem cell pool and may provide a mechanism for restricting excess organ growth. Thrombin is the major serine protease that cleaves OPN, giving rise to a 24-kDa and a 45-kDa fragment. The 45-kDa fragment has multiple functional advantages in processes such as cell attachment, migration, and spreading, through binding to a9b1 and a4b11. Our present finding that treatment of factor VIII KO recipients of pig embryonic spleen exhibit a deuced implant size, similarly to that found in the non-hemophilic recipients, strongly indicates that thrombin likely exerts its regulatory effect through cleavage of OPN .

While studies on the HSC niche in the bone marrow are relatively well advanced[33]-[35]very little is known regarding the stem cell niche in the embryonic pig tissue used for transplantation in our assays. The new insights on the role of thrombin and tcOPN as well as the well characterized binding of the latter to the integrins α9β1 and α4β11, could facilitate our ability to identify and localize stem cell - niche interactions which might occur in non –hematopoietic organs and tissues.

However, other possibilities that might underline the inhibitory activity exerted by tcOPN, including potential effect on the vasculature should be considered.

In conclusion, our data suggest a novel role for factors of the coagulation cascade in organ size control. In particular, we were able to pinpoint thrombin and its substrate OPN as the direct players.

This surprising finding may not only offer new means to enhance or reduce the final size of implanted xenogeneic embryonic tissues, but might also adds a novel insight into the mysterious question of organ size control in mammals. Clearly, further studies investigating the role of the coagulation cascade factors in more physiological settings, and in particular their potential ability to affect normal tissue stem cells under extreme growth stimuli, are warranted.

## Materials and Methods

### Animals

All animals were maintained under conditions approved by the Institutional Animal Care and Use Committee at the Weizmann Institute. The study protocol was approved by the ethics committees at Kibbutz Lahav and the Weizmann Institute. In these experiments, 8–10 week old immune deficient NOD-SCID mice and NOD-SCID Factor VIII KO mice (Weizmann Institute Animal Breeding Center, Rehovot, Israel) or RAG−/− mice and RAG−/− FVIII KO mice were used as hosts for the transplantation studies. To obtain immunodeficient hemophilic mice (designated Factor VIII KO–SCID), FVIII-deficient mice were crossed with SCID or RAG−/− mice, as was previously described[11].

For G-CSF studies, 8 to 10 week old immune competent C57BL mice and C57BL hemophilic (C57BL Hem F8) mice were used. All mice were kept in small cages (up to five animals per cage) and fed sterile food.

Pig embryos were obtained from the Lahav Institute of Animal Research (Kibbutz Lahav, Israel). Pregnant sows were operated on at embryonic day 28 (E28) for the harvest of liver, and at E42 for the harvest of pancreas and spleen, under general anesthesia. Warm ischemia time was less than 10 minutes and the embryos were transferred to cold PBS. Spleen, pancreas and liver precursors for transplantation were extracted under a light microscope and were kept in sterile conditions at 4°C in RPMI 1640 (Biological Industries, Beit HaEmek, Israel) prior to transplantation. Cold ischemia time until transplantation was less than 2 hours. Mouse embryos were obtained from C57BL/6 pregnant female mice. Pregnant mice were operated on at embryonic day 15 (E15), to remove fetal spleen tissue, and at day 16 (E16) for liver and pancreatic tissues, under general anesthesia. Warm ischemia time was less than 10 minutes, and the embryos were transferred to cold PBS. Spleen, pancreas and liver precursors for transplantation were extracted under a light microscope and were kept in sterile conditions at 4°C in RPMI 1640 (Biological Industries, Beit HaEmek, Israel) prior to transplantation. Cold ischemia time until transplantation was less than 2 hours.

### Transplantation Procedure

Transplantation of pig precursors was completed as previously described[36]. Briefly, transplantation of the embryonic precursors was performed under general anesthesia (2.5% 2,2,2-Tribromoethanol in PBS, 10 ml/kg intraperitoneally). Host kidney was exposed through a left lateral incision. A 1.5-mm incision was made at the caudal end of the kidney capsule and donor precursors were grafted under the kidney capsule in fragments 1–2 mm in diameter.

### Morphometric Analysis

Porcine E42 spleen and pancreatic grafts were formalin fixed and embedded in paraffin 3 months following transplantation. Consecutive 40 µm sections were cut and stained with pig specific anti ki67 antibody. The areas of interest were quantified using the Image Pro program (Media Cybernetics).

### ELISA Measurements of Pig Insulin and Albumin

A porcine/human insulin kit (Catalog No. K6219, DAKO), in which the primary pig anti-insulin antibody does not cross-react with mouse insulin, was used, according to the manufacturer's instructions, to follow pig insulin levels. Pig albumin in mouse serum was measured by a standard ELISA using primary affinity purified goat anti-pig albumin antibody (human, mouse, and bovine adsorbed), and secondary pig-specific horseradish-peroxidase conjugated antibody (Catalog Nos. A100–210A and A100–210P, Bethyl).

### Histochemistry

Histochemistry included hematoxylin/eosin (H&E) and periodic acid/Schiff (PAS) staining. For immunohistochemical labeling, the following antibodies were used: Goat anti-pig albumin antibody (Bethyl Laboratories, Montgomery, TX), rabbit anti-human glucagon (DAKO), guinea pig anti-rabbit insulin (DAKO), mouse anti porcine CD31 (Serotec, Enco Scientific Services Ltd Israel) mouse anti porcine vimentin (clone V9) (Dako) and mouse anti-human ki67 (clone MIB-1) (Dako). Paraffin sections (4 µM) were xylene deparaffinized and rehydrated. Endogenous peroxidase was blocked with 0.3% H2O2 in 70% methanol for 10 minutes. Antigen-retrieval procedures were performed according to the manufacturer's (DAKO) instructions. After blocking, both paraffin sections and 6-µM cryosections were incubated with specific first antibody for 60 minutes. Detection of antibody binding was performed by using the following secondary reagents: DAKO peroxidase EnVision system for the detection of mouse and rabbit antibodies, and Sigma biotinylated anti-goat antibody (followed by extra avidin peroxidase reagent) for goat antibodies. In all cases, diaminobenzidine was used as a chromogen. Immunofluorescence protocols were applied using secondary antibodies: Donkey anti mouse Texas red (Jackson), and donkey anti rat conjugated CY2 or Texas red (Jackson).

### Treatment with Inhibitors

Enoxaparine (Clexane 20 mg/0.2 ml, Rhone-poulenc, France) was used at a dosage of 200 µg/mouse, (dissolved in PBS) and 0.2 ml of the final solution was injected subcutaneously into each mouse once a day. Dabigatran etexilate (Boehringer Ingelheim Pharma KG, Biberach, Germany) was administered orally at a dosage of 30 mg/kg. A final volume of 0.3 ml dissolved in DDW was administered daily. PAR1 and PAR4 antagonists: The palmitoylated peptides: pal-RCLSSSAVANRS (PAR1 antagonist) and pal-SGRRYGHALR (PAR4 antagonist) were prepared by solid-phase peptide synthesis using in situ neutralization/HBTU by Hadar Biotec, Israel. The mice were treated by vehicle control, or with PAR1antagonist or PAR4 antagonist 0.5 mg/kg, intraperitoneally on daily basis. Thrombin cleaved form of Osteopontin (tcOPN) was produced as previously described[37], administration of tcOPN was twice daily by orbital vein injection through all transplantation period.

### Statistical Analysis

Comparisons between groups were evaluated by the Student' test. Data were expressed as mean±SD, and were considered statistically significant at p values of 0.05 or less.
